# RECEIVED SOCIAL SUPPORT SATISFACTION MAY INFLUENCE THE PERCEIVED STRESS-COGNITION ASSOCIATION IN BLACK AMERICANS

**DOI:** 10.1093/geroni/igae098.0765

**Published:** 2024-12-31

**Authors:** DeAnnah Byrd, David Coon, Roland Thorpe, Jr., Keith E Whitfield

**Affiliations:** Arizona State University, Phoenix, Arizona, United States; Arizona State Unviersity, Phoenix, Arizona, United States; Johns Hopkins University, Baltimore, Maryland, United States; University of Nevada Las Vegas, Las Vegas, Nevada, United States

## Abstract

Previous research suggests that perceptions of stress shape cognitive health. Social support has been shown to be protective. Yet, it is not clear whether satisfaction with support is also beneficial. The aim of this study was to determine whether perceived stress is associated with cognitive changes over time and if satisfaction with receipt of emotional or instrumental support acts as a moderator in a sample of older Blacks 48 to 95 years of age, who were enrolled in the Baltimore Study of Black Aging—Patterns of Cognitive Aging and interviewed at two time points, approximately 3 years apart (N = 450). Cognition included 5 domains: working memory, processing speed, verbal memory, vocabulary, and inductive reasoning. Social support satisfaction was measured by asking participants “How satisfied are you with the emotional or instrumental support that you get from your family and friends in terms of how they make you feel and how much it helps you?” Results from the linear regression model showed no direct effect of stress on cognitive decline. Satisfaction with instrumental support marginally (p <.10) modified the relationship between stress and working memory at follow-up but was not related to any other cognitive domains, adjusting for age, sex, education, depressive symptoms, comorbidities, and baseline cognition. Findings demonstrate the joint effects of stress and social support satisfaction on cognition, highlighting the need for more research documenting protective factors for domain-specific cognitive decline. This approach provides a basis for developing cognitive interventions and support networks for this population of Black Americans.

